# Development and validation of a smartwatch algorithm for differentiating physical activity intensity in health monitoring

**DOI:** 10.1038/s41598-024-59602-6

**Published:** 2024-04-25

**Authors:** Daixi Chen, Yuchen Du, Yuan Liu, Jun Hong, Xiaojian Yin, Zhuoting Zhu, Jingjing Wang, Junyao Zhang, Jun Chen, Bo Zhang, Linlin Du, Jinliuxing Yang, Xiangui He, Xun Xu

**Affiliations:** 1 Department of Ophthalmology, Shanghai General Hospital, Shanghai Jiao Tong University School of Medicine, National Clinical Research Center for Eye Diseases, Center of Eye Shanghai Key Laboratory of Ocular Fundus Diseases, Shanghai Engineering Center for Visual Science and Photomedicine, Shanghai, 200080 China; 2 Shanghai Eye Disease Prevention and Treatment Center, Shanghai Eye Hospital, School of Medicine, Tongji University, National Clinical Research Center for Eye Diseases, Shanghai Engineering Research Center of Precise Diagnosis and Treatment of Eye Diseases, Shanghai, 200030 China; 3 Key Laboratory of Adolescent Health Assessment and Exercise Intervention of the Ministry of Education, East China Normal University, Shanghai, 200241 China; 4 College of Economics and Management, Shanghai Institute of Technology, Shanghai, 201418 China; 5 Centre for Eye Research Australia, Ophthalmology, University of Melbourne, Melbourne, VIC 3010 Australia

**Keywords:** Physical activity, Myopia control, Machine learning algorithm, Smartwatch, Epidemiology, Classification and taxonomy, Computational models, Predictive medicine, Eye diseases

## Abstract

To develop and validate a machine learning based algorithm to estimate physical activity (PA) intensity using the smartwatch with the capacity to record PA and determine outdoor state. Two groups of participants, including 24 adults (13 males) and 18 children (9 boys), completed a sequential activity trial. During each trial, participants wore a smartwatch, and energy expenditure was measured using indirect calorimetry as gold standard. The support vector machine algorithm and the least squares regression model were applied for the metabolic equivalent (MET) estimation using raw data derived from the smartwatch. Exercise intensity was categorized based on MET values into sedentary activity (SED), light activity (LPA), moderate activity (MPA), and vigorous activity (VPA). The classification accuracy was evaluated using area under the ROC curve (AUC). The METs estimation accuracy were assessed via the mean absolute error (MAE), the correlation coefficient, Bland–Altman plots, and intraclass correlation (ICC). A total of 24 adults aged 21–34 years and 18 children aged 9–13 years participated in the study, yielding 1790 and 1246 data points for adults and children respectively for model building and validation. For adults, the AUC for classifying SED, MVPA, and VPA were 0.96, 0.88, and 0.86, respectively. The MAE between true METs and estimated METs was 0.75 METs. The correlation coefficient and ICC were 0.87 (*p* < 0.001) and 0.89, respectively. For children, comparable levels of accuracy were demonstrated, with the AUC for SED, MVPA, and VPA being 0.98, 0.89, and 0.85, respectively. The MAE between true METs and estimated METs was 0.80 METs. The correlation coefficient and ICC were 0.79 (*p* < 0.001) and 0.84, respectively. The developed model successfully estimated PA intensity with high accuracy in both adults and children. The application of this model enables independent investigation of PA intensity, facilitating research in health monitoring and potentially in areas such as myopia prevention and control.

## Introduction

Myopia is the most common eye condition worldwide, is most often characterized by a blurring of objects viewed at a distance. This blurring is typically a result of an abnormal elongation of the eyeball, causing the refractive image formed by the cornea and the lens to fall in front of the retina, rather than directly on it^1,2^. Generally, myopia is commonly defined as a spherical equivalent (SE) ≤ − 0.50 diopters (D)^3^. Recently, considerable attention has been given to the protective role of outdoor time in the onset of myopia^4–6^. However, the impact of physical activity (PA) on myopia remains a subject of debate due to the confounding effects of time spent outdoors^7–12^. Consequently, an device-based measurement is needed to quantify PA independently of outdoor time.

Although various devices like accelerometers are available for physical activity (PA) assessment, they often fail to accurately differentiate between indoor and outdoor activity states^13,14^. Our smartwatch technology innovatively integrates light intensity sensing with triaxial accelerometer data, enabling more precise differentiation. Our team has developed an algorithm that segments indoor and outdoor states based on raw data from smartwatch^15^. This study aims to develop a model for segmenting and estimating the intensity of PA. By combining the algorithms for distinguishing indoor and outdoor states with the PA segmentation developed in this study, it is possible to differentiate between indoor and outdoor PA and intensities. This capability is crucial for understanding the relationship between PA and myopia and holds significant value for broader health monitoring research.

In recent years, machine learning (ML) techniques have been increasingly applied in health monitoring, including the assessment of PA levels^16–18^. These techniques have demonstrated significant potential in extracting meaningful insights from complex health data, leading to more accurate and device-based measurements in various health-related research fields^19,20^. Among the various ML algorithms that have been explored for the segmentation of PA from raw accelerometer data, the support vector machine (SVM) algorithm stands out for its application in our study^21,22^. We intend to utilize SVM due to its suitability for segmenting PA, benefiting from its robust performance in both binary and multi-class problems^16–18^. This is supported by its capability to handle large datasets effectively and its versatility in managing linear and non-linear data structures. The kernel functions of SVM, which facilitate linear separation in higher-dimensional space, are particularly advantageous for dealing with the high-dimensional data encountered in our domain^23^. Additionally, a review of 19 studies assessing the accuracy of machine learning algorithms in classifying PA indicated that SVM outperformed other techniques such as Artificial Neural Networks (ANN), Decision Trees (DT), and Naive Bayes (NB). Moreover, SVM was identified as the most frequently employed ML algorithm in the studies included in the review^18^.

In this study, we aim to apply the ML ﻿algorithms to develop a model for classifying and estimating PA in young adults and children with information from participants and PA data of our smartwatch. Meanwhile, the accuracy and agreement performance of the model was validated.

## Methods

### Participants

Two groups of participants were recruited for the study, including 24 adults aged 21–34 years and 18 children aged 9–13 years. Among the participants, there were 13 (54.2%) adult males and 9 (50.0%) male children. ﻿Subjects were excluded if any medical condition that may inhibit physical activity or affect metabolic rate, such as cardiovascular disease, metabolic disorder, skeletal motor system disease, and so on. For children, the subjects older than 13 years were excluded. All the participants must be able to exercise without assistance and complete the required activity trials. The study was conducted according to the tenets of the Declaration of Helsinki, with ethics approval from Institutional Review Board of Shanghai General Hospital, Shanghai Jiao Tong University. Informed consent was obtained from parents or guardians and verbal assent was obtained from children.

### Study design

**Figure 1:**
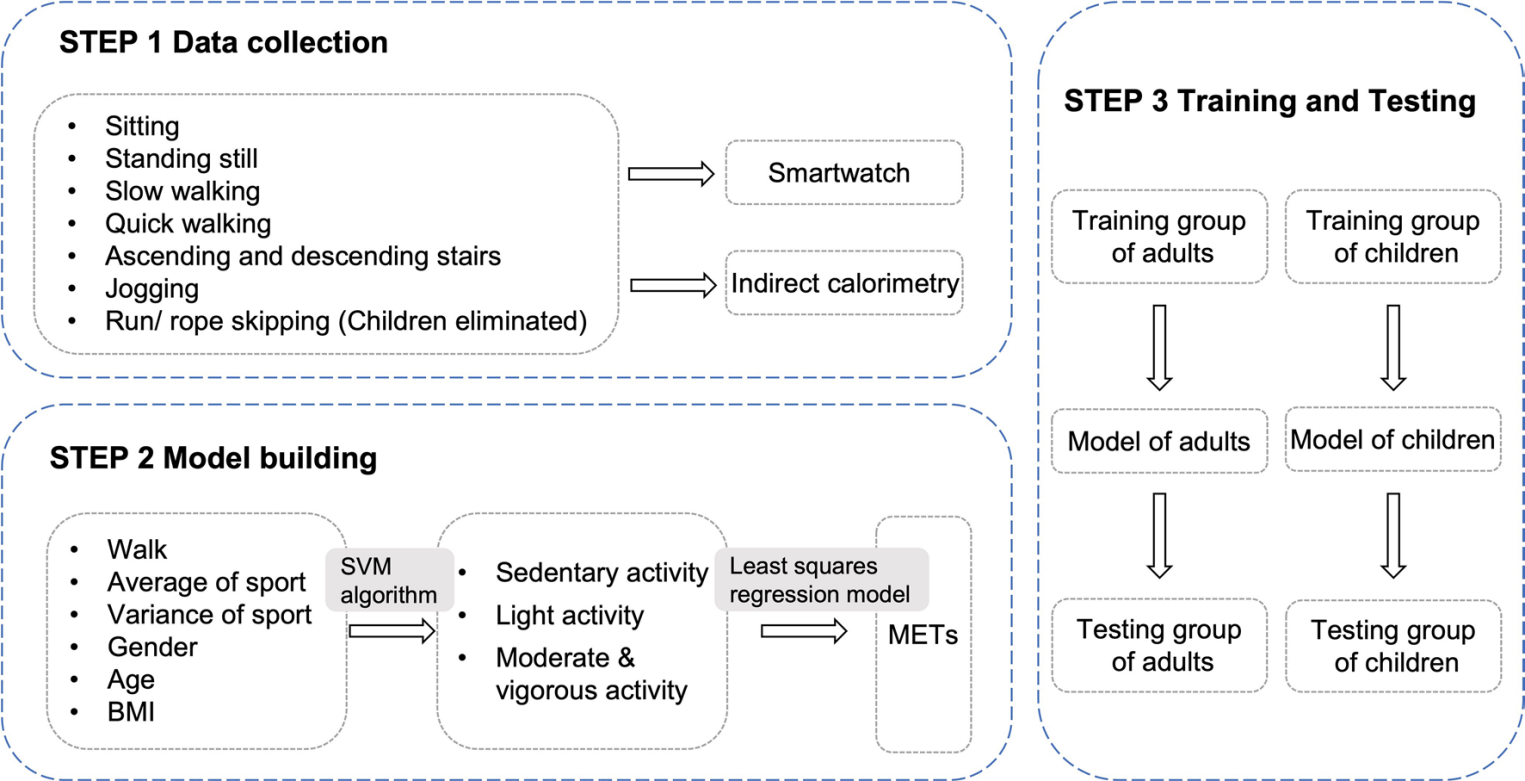
Flowchart of study design. In step 1, the data of smartwatch and indirect calorimetry of adults and children were collected. In step 2, the support vector machine (SVM) algorithm and the least squares regression model were applied for the metabolic equivalent (MET) estimation. In step 3, two models were built and validated for the 2 testing groups.

﻿Personal information including age, height, weight, and birth were collected. ﻿BMI was calculated as ﻿weight (kg) divided by height squared (m^2^). For the activity types, Welk suggested that the activities ﻿design for ﻿accelerometry-based calibration should include a ﻿variety of activity levels and emphasize the most common movements. Besides, the free-living activities were﻿ preferable to lab-based treadmill movements^24^. Accordingly, the selected activities ranged from ﻿sedentary to vigorous intensity, included 7 usual types, and were conducted in living environment. The ﻿description of each activity is presented in Table 1. For adults, participants completed the whole 7 ﻿sequences of activities. For children, observing that some children exhibited more energy expenditure than anticipated when instructed to jog, combined with the consideration of their varied physical capabilities and adherence, we decided to eliminate jogging from the vigorous activities. Each participant performed the activities from ﻿sedentary to ﻿the most vigorous intensity in a standardized manner (Step 1 in Fig. 1). Each activity trial was conducted for 14 to 16 min, with data﻿ on the first 3 min and the last 1 min excluded from analysis. Before performing the activities, participants wore the smartwatch ‘Mumu’ on the wrist and equipped with the ﻿indirect calorimetry machine.

**Table 1:** Description of each activity trial.

Activity type	Intensity	Description
Sitting	Sedentary	Seated in a chair at a desk and using mobile phones or reading books
Standing still	Sedentary	Standing ﻿quietly. Instructed not to move around
Slow walking	Light	Walking for pleasure, ﻿at a self-selected slow speed
Quick walking	Moderate	Walking for exercise, ﻿at a self-selected quick speed
Ascending and descending stairs	Moderate	Walking up and down stairs at a self-selected usual speed
Jogging	Vigorous	Jogging at a self-selected usual speed
Run/rope skipping	Vigorous	Running at a self-selected usual speed or jumping rope at moderate pace, 100–120 skips/min, 2-foot skip

### Smartwatch

**Figure 2:**
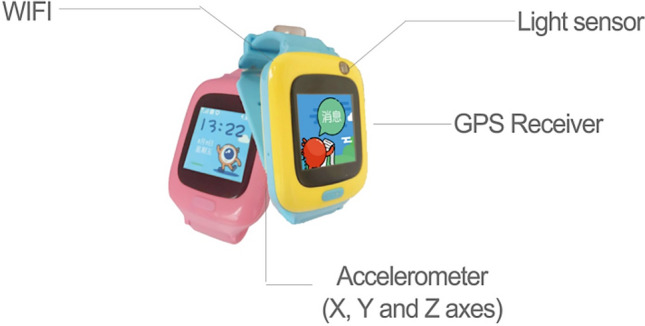
The construction of the smartwatch.

Our team designed and developed a smartwatch which was equipped with a 3 axes accelerometer, a light sensor, and a GPS receiver (Fig. 2)^25^. The accelerometer consists of 3 axes that indicate the X, Y, and Z axes in space and through filtering, peak-valley detection, and removing interference, converts the data into counting steps, average of PA, and variance of PA. The light sensor samples luminance and ultraviolet intensity at 20-s intervals. The built-in GPS receivers are used for receiving satellite signals and collecting data on the longitude and latitude of the location. The smart watch samples data once a minute. One piece of data consists of: time (year/month/day/00:00:00), average of PA, variance of PA, count of steps, 3 data points of luminance (lx), 3 data points on ultraviolet light intensity, and wearing status. The above data were uploaded by the mobile terminal to a software platform, that was developed for collecting, analyzing, and counting the data.

### Indirect calorimetry

Previous research often utilized metabolic equivalent (MET) values to estimate the intensity of PA, where one MET is defined as the amount of oxygen consumed while at rest, equivalent to 3.5 ml O_2_ per kg body weight per minute^17,26,27^. Several studies estimating PA consider device-based indirect calorimetry as the gold standard and categorize PA into three distinct types (SED: 0–1.49 METs; LPA: 1.50–2.99 METs; MPA: 3.00–5.99 METs; VPA: ≥ 6.00 METs)^28–33^. In our study, the METs were measured by a portable metabolic analyzer K5 (COSMED, Rome, Italy). The K5 consisted of a wearable ﻿miniaturized analyzer device and the attached mask. The mask was placed to cover the nose and mouth of participants for ﻿measuring the volume of inspired and expired air, and the analyzer system could pair the computer software for outputting the results. The device weighted about 900 g, was worn on the back of participants during activity intensity measurement. Calibration of the K5 was conducted using standard gases (﻿4% CO_2_ and 16% O_2_) before each test. The resting ﻿and exercise ﻿oxygen uptake was measured and the METs were derived by oxygen consumption of each activity dividing by REE. Evidence has been proved that the COSMED K5 can provide reliable and valid measures of metabolic variables ﻿over a range of PA intensities^34–36^. Additionally, the COSMED has been used for accelerometer calibration protocols in different age of participants^37–40^.

### Data ﻿processing

The data were excluded for the training set and testing set if the smartwatch ﻿failed to record the PA information or if EE were not in a stable state. The measured METs by indirect calorimetry were set as gold standard for model building in training set and evaluation in testing set. Participants were randomly assigned into either the training set or the testing set at about a proportion of 70% and 30% in adults and children, respectively. The data of ‘average of PA, ‘variance of PA, and ‘count of steps’ from the smartwatch, and gender, BMI were used to estimate METs. Average measured METs in each minute were calculated to match the smartwatch data.

For the ‘average of PA’ (PA_avg), it was computed from the acceleration values measured by the three-axis accelerometer, which samples data at a frequency of 2 times per second, resulting in 120 readings per minute. For each axis (x, y, z), we calculated the average maximum change in acceleration per minute. For instance, the calculation for the x-axis would be: PA_avg(x) = (x 2 − x 1) + (x 3 − x 2) + ⋯ + (x 120 − x 119)/119. This process was similarly conducted for the y and z axes. The final ‘average of PA’ value was determined as the maximum among these three axis values. For the ‘variance of PA’ (PA_s), after identifying the axis with the maximum average change in acceleration, we then calculated the variance of the acceleration changes relative to this average. The formula for this was: PA_s = ((x 2 − x 1) − PA_avg)^2^ + ((x 3 − x 2) − PA_avg)^2^ + ⋯ + ((x 120 − x 119) − PA_avg)^2^/119. Additionally, the smartwatch's three-axis accelerometer automatically recorded the ‘count of steps’ based on the user's physical activity.

### Model building

﻿This study initially classified the PA intensities into SED, LPA, and MVPA using the ﻿support vector machine (SVM) algorithm followed by METs estimated using a least squares regression model with measured METs. The SVM ﻿algorithm was adopted to solve the PA intensity classification problem. The data on ‘average of PA’, ‘variance of PA’, and ‘count of steps’ was primarily transferred into log-scale to be readily used for the following classification and modelling. The SVM algorithm established a set of hyperplanes to separate the instances into different categories with respect to the PA intensity. The SVM model with a linear kernel was employ as the classification model. The SVM model was trained on the training set. During the training procedure, the grid-search strategy and the fivefold cross validation was used to automatically optimize the parameters of SVM. The performance of the learned model was validated in the test set. ﻿For instances of each PA intensity, the least squares regression was used to build a specific model to estimate METs of each instance. As a result, 3 models were learned for each PA intensity category. The estimated METs were eventually obtained through the models (Step 2 and 3 in Fig. 1).

### Statistical analysis

﻿Normality of data was ﻿assessed using ﻿a Shapiro–Wilk test. Significance level was set at *p* ≤ 0.05. The testing set were analyzed to evaluate the METs estimation model in adults and children. To evaluate the classification accuracy for discriminating between 2 populations, the estimated METs were set as binary variables by intensity categories of PA. The sensitivity, specificity, and ﻿the area under the ROC curve (AUC) were calculated. For the accuracy of estimated METs, the mean absolute error (MAE), the mean absolute percentage error (MAPE), and the root mean square error (RMSE) were employed. Additionally, ﻿Bland–Altman plots and ﻿intraclass correlation (ICC) were used to ﻿assess the agreement between true METs and the estimated METs. The relationship of the true METs and the estimated METs were ﻿evaluated by Spearman ﻿correlations. Data were analyzed using SPSS version 26.0 (SPSS, Inc., Chicago, IL, USA).

### Ethics approval and consent to participate

The study was approved by the ethics committee of the Shanghai General Hospital.

### Consent for participation

Informed consent was obtained from parents or guardians of the children. Also informed consent was obtained from adult participants.

## Results

### Participants’ characteristics and activity trials

A total of 1790 and 1246 pieces of data for adults and children respectively were included for model building and validation. The body mass index (BMI) was 21.0 ± 2.9 kg·m^−2^ for adults and 20.2 ± 3.2 kg·m^−2^ for children (Table 2). The training set and testing set were similar in gender, age, height, weight, and BMI. The mean and SD of measured METs for the different type of PAs in all adults and children are displayed in Table 3. The PA intensity ranged from SED to VPA. According to the measured METs, the sitting and standing still trials were considered SED intensity; the slow walking trial was considered LPA intensity; MPA intensity consisted of ascending and descending stairs; and trials of jogging and run/rope skipping were taken for VPA. However, METs of some activity trials could not be categorized into one intensity range because of the massive individual variability in EE in each activity trial.

**Table 2:** Characteristics of included participants.

	Training set	Testing set	Total
Gender (male/female)			
Adults	10/7	3/4	13/11
Children	7/6	2/3	9/9
Age (years)			
Adults	25.3 ± 2.8	23.9 ± 1.9	24.9 ± 2.6
Children	12.2 ± 1.1	12.4 ± 0.5	12.3 ± 1.0
Height (cm)			
Adults	169.4 ± 7.2	168.9 ± 7.9	169.2 ± 7.2
Children	159.2 ± 11.4	163.9 ± 6.1	160.5 ± 10.3
Weight (kg)			
Adults	61.6 ± 7.6	62.0 ± 11.6	61.7 ± 8.7
Children	52.3 ± 14.4	53.5 ± 6.1	52.6 ± 12.5
BMI (kg m^−2^)			
Adults	21.5 ± 2.6	21.6 ± 2.3	21.0 ± 2.9
Children	20.3 ± 3.6	19.9 ± 1.8	20.2 ± 3.2
Data points			
Adults	1342	448	1790
Children	862	384	1246

Values are presented as mean ± SD.

**Table 3:** Mean and SD values of measured METs for each activity in all adults and children.

Activity type	Adults		Children	
	Mean	SD	Mean	SD
Sitting	1.01	0.13	1.04	0.19
Standing still	1.11	0.19	1.19	0.42
Slow walking	2.23	0.62	2.52	0.80
Quick walking	3.66	1.14	3.56	1.32
Ascending and descending stairs	5.30	1.89	6.01	2.18
Jogging	6.41	2.38	Not available	Not available
Run/ rope skipping	9.40	3.74	6.68	2.58

### Accuracy of PA classification in testing set

**Figure 3:**
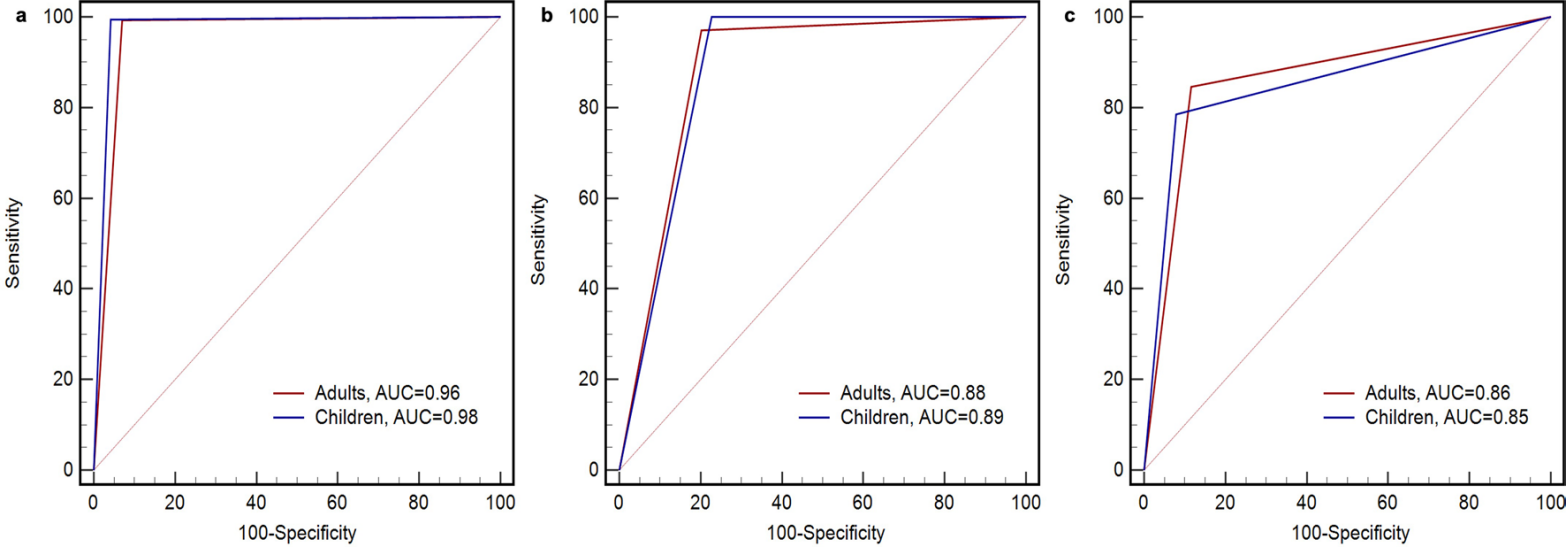
ROC curve of the algorithm for classifying SED (**a**), MVPA (**b**), and VPA (**c**) in adults and children.

Figure 3 provides the ROC curves of the algorithm for classifying different intensities of PA in adults and children. In adults, the model showed excellent sensitivity and specificity for classifying the SED intensity (99.4% and 92.9%, respectively). For classifying MVPA and VPA, the model displayed good sensitivity (97.1% and 84.6%, respectively) as well, while the specificity for classifying VPA (88.4%) was higher than that of MVPA (79.8%). When evaluating the accuracy of the model, the Youden indexes for classifying SED and LPA or above (0.92 and 0.92, respectively) were higher than that of MVPA and VPA (0.77 and 0.73, respectively). Additionally, the AUC displayed ﻿almost perfect accuracy for the classification of SED (AUC = 0.96, 95% CI 0.94–0.98). The discrimination of the model for MVPA and VPA (AUC = 0.88, 95% CI 0.85–0.92) and AUC = 0.86, 95% CI 0.81–0.92 respectively) were less accurate than SED, but nonetheless of acceptable accuracy.

For children, the model for SED exhibited ﻿excellent ﻿classification accuracy (Youden indexes = 0.95; AUC = 0.98, 95% CI 0.96–0.99), with high sensitivity and specificity (95.7% and 99.5%, respectively). For classifying MVPA, the model showed reasonable sensitivity (77.2%) with perfect specificity (100.0%). For VPA, the model provided excellent sensitivity (92.1%) and fair specificity (78.6%). Consequently, the Youden indexes were fair for distinguishing MVPA and VPA (0.77 and 0.71, respectively). The AUC indicated that the model was good for classifying MVPA and VPA (0.89, 95% CI 0.85–0.92 and 0.85, 95% CI 0.76–0.94 respectively). Additionally, the AUC between adults and children for distinguishing PA intensities displayed no significant difference. Table 4 presents the sensitivity, specificity, Youden index, and AUC for the model discriminating PA intensities for adults and children.

**Table 4:** The sensitivity, specificity, and area under the ROC curve of the algorithm for classifying PA intensities in adults and children.

	SED	MVPA	VPA
Adults			
Sensitivity, 95% CI	99.4, 98.2–100.0	97.1, 94.9–99.4	84.6, 76.6–92.6
Specificity, 95% CI	92.9, 89.3–95.6	79.8, 74.7–84.9	88.4, 85.1–91.6
Youden index	0.92	0.77	0.73
AUC, 95% CI	0.96, 0.94–0.98	0.88, 0.85–0.92	0.86, 0.81–0.92
Children			
Sensitivity, 95% CI	95.7, 92.9–98.6	100.0, 100.0–100.0	92.1, 89.3–94.9
Specificity, 95% CI	99.5, 98.5–100.0	77.2, 72.1–82.3	78.6, 63.4–93.8
Youden index	0.95	0.77	0.71
AUC, 95% CI	0.98, 0.96–0.99	0.89, 0.85–0.92	0.85, 0.76–0.94
*p* value ^a^	0.20	0.94	0.81

^a^*p* value for AUC comparison between adults and children classifying PA intensities.

### Accuracy and precision of METs estimation in testing set

In adults, the median of estimated METs was higher than true METs (3.84 METs vs. 2.74 METs), while the opposite result was obtained in children (1.11 METs vs. 1.37 METs; Table 5). The overall MAEs were both within 1 METs in adults and children (Adult = 0.75 ± 0.95 METs and Children = 0.80 ± 0.90 METs). Therefore, the accuracy of MET estimation in adults was slightly higher than in children, as further demonstrated by MAPEs (Adult = 25.05% vs. children = 29.48%). Moreover, the RMSE for adults and children were 1.21 ± 2.04 and 1.21 ± 1.59 respectively.

**Table 5:** The true METs, estimated METs, and MAE, MAPE, and RMSE between measured and estimated METs in adults and children.

	Adults	Children
Estimated METs	3.84, 1.07–5.89	1.11, 1.02–5.01
True METs	2.74, 1.12–5.61	1.37, 1.06–4.44
MAE	0.75 ± 0.95	0.80 ± 0.90
MAPE, %	25.05 ± 39.09	29.48 ± 33.30
RMSE	1.21 ± 2.04	1.21 ± 1.59

### Correlation and agreement between true METs and estimated METs in testing set

**Figure 4:**
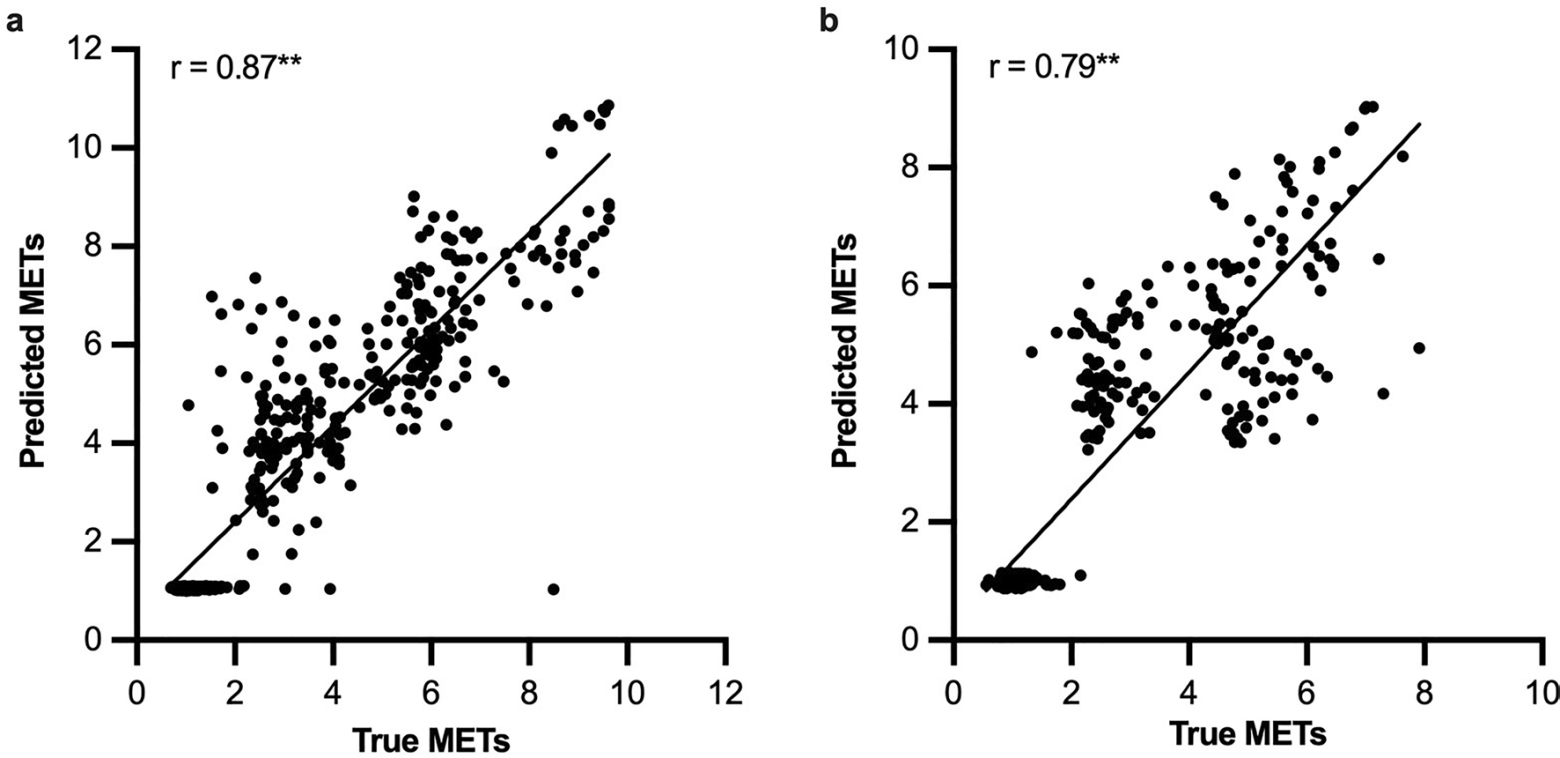
Correlation plots between true METs and estimated METs in adults (**a**) and children (**b**). The Spearman coefficient is 0.87 (**p < 0.001, estimated METs compared with true METs) for adults and 0.79 (**p < 0.001, estimated METs compared with true METs) for children, respectively.

**Figure 5:**
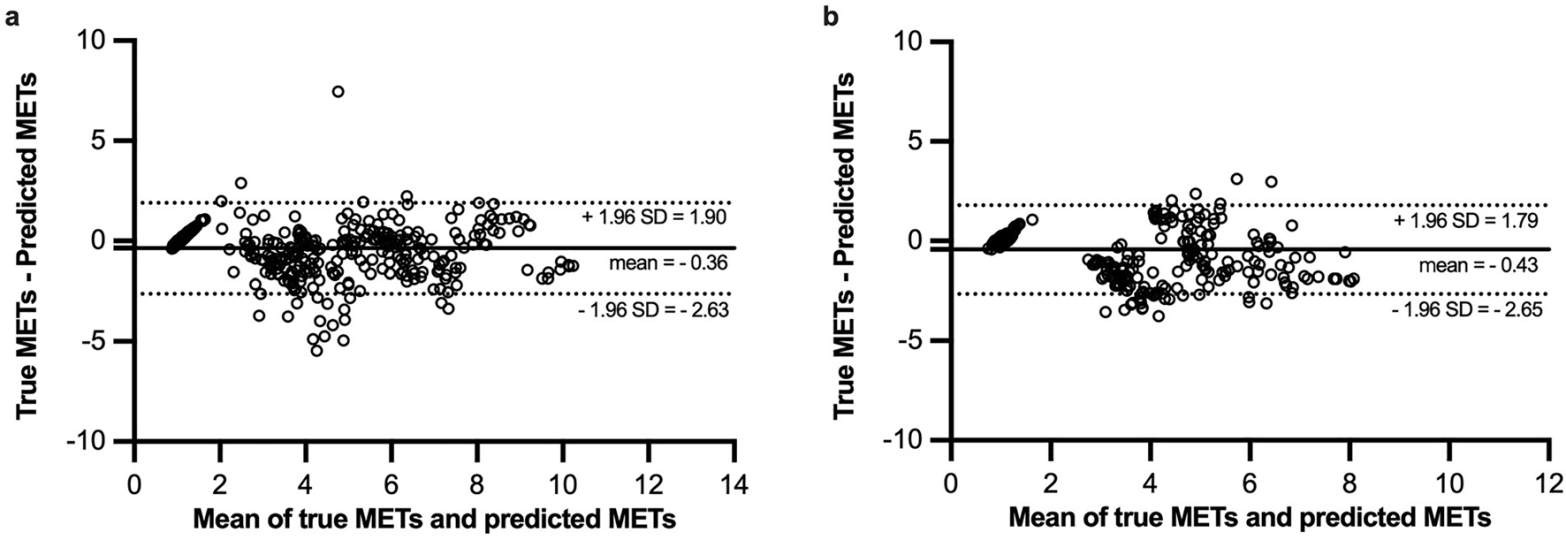
Agreement between true METs and estimated METs (Bland–Altman plot) in adults (**a**) and children (**b**). (**a**) The bias is − 0.36 (solid line), the 95% limit of agreement (LoA) are − 2.63 and 1.90 (dotted line). (**b**) The bias is − 0.43 (solid line), the 95% limit of agreement (LoA) are − 2.65 and 1.79 (dotted line).

The correlation was good for adults (Spearman coefficient = 0.87, *p* < ﻿0.001; Fig. 4a) and ﻿reasonable for children (Spearman coefficient = 0.79, *p* < ﻿0.001; Fig. 4b). For the agreement between true METs and estimated METs, the mean differences were both low in adults and children (− 0.36 METs and − 0.43 METs, respectively; Fig. 5a). The ﻿95% limit of agreement (LoA) were − 2.63 to 1.90 for adults and − 2.65 to 1.79 for children (Fig. 5b). Additionally, the ICC was 0.89 and 0.84 for adults and children, respectively.

## Discussion

Our smartwatch, equipped with a tri-axial accelerometer, is primarily designed to accurately measure various intensities of PA. This study developed and validated a new model to classify PA intensities, based on estimated METs for adults and children. The model exhibited excellent accuracy (AUC = 0.96–0.98) for classifying SED and LPA or above and good accuracy for distinguishing MVPA and VPA (AUC = 0.85–0.89) in young adults and children. Moreover, the estimated METs ﻿exhibited good accuracy (MAE = 0.75–0.80 METs) and agreement (ICC = 0.84–0.89) when compared with measured METs. Equipped with a highly accurate PA estimation model and an outdoor state discrimination algorithm developed by our team formerly^15^, the smartwatch could be applied to large-scale research, to analyze the role of PA and the outdoor time for myopia protection independently.

We collected data on a wide range of PA intensities in a controlled setting, alongside gold-standard measurements for validation. Additionally, we utilized SVM algorithms to classify PA intensities, optimizing the algorithm's performance. Comparing the EE in this study with that of prior research, the measured METs were similar in each type of activity trial. The EE of SED intensity in adults and children, ranged from 1.01 to 1.19 METs in the current study, similar to the 1.0–1.3 METs recorded in the former research^40–42^. For LPA, the EE was 2.23–2.52 METs compared to 2.2–3.3 METs in the previous studies^40,43^. For MPA, the EE ranged from 3.56–6.01 METs, while previous studies recorded 3.0–7.6 METs^40,44,45^. Finally, for VPA intensity, the EE was 6.41–9.40 METs in our study and 6.5–9.4 METs in the previous studies^40,44,45^. Some discrepancies of the METs between our study and previous studies may derive from the different characteristics of subjects. However, overall, the measured METs displayed the consistent reliability for PA quantification in the current study.

To compare with previous studies, among the device-based approaches applied in myopia research, several studies had assessed the accelerators for measuring PA intensity. The CHAMPS Eye Study^9,11^ used the ﻿GT3X + accelerometer (ActiGraph, Florida, FL, USA) to measure PA. The cut-off points for different intensity levels have been validated by previous studies^41,42^. For SED, both our model and the previous studies showed excellent classification accuracy (AUC = 0.96–0.98 for adults and children in the present study vs. AUC = 0.90–0.98 in the previous study)^41,42^. Although the accuracy of MVPA classification was lower than that for SED discrimination (AUC = 0.88–0.89 in adults and children), in comparison to the previous studies the accuracy was equivalent (AUC = 0.85–0.90)^41,42^. In terms of VPA, our model demonstrated comparable discrimination accuracy (AUC = 0.85–0.86 for adults and children in the current study) when considered alongside AUC values reported in previous studies (AUC = 0.83–0.84)^41,42^. Additionally, it seems that our model shows better specificity than that of the previous studies (77.2–99.5% in present study vs. 66–73% in previous studies), and the sensitivity of our model was as good as the previous studies (78.6–100.0% in present study vs. 86–92% in previous study)^46,47^. Overall, our model for PA classification displays good accuracy and well potential for the myopia research.

This is the first study estimating METs in children below 13 years of age, with results showing high accuracy and reliability. In addition, the estimation model for adults in the current study was as good as previous studies^26,27,43,44,48^. Recently, Wu et al.^26^ presented a new equation to improve adult METs estimation using the activPAL device. The overall MAPE for the new equation and the ﻿built-in equation was 14.6% and ﻿23.4%, respectively. Another study found a MAPE of 18% for adults in the comparison between ﻿activPAL and indirect calorimetry^27^. Our MAPE for adults was 25.05%. This discrepancy may be explained by the differing conditions under which the respective studies are performed, with former studies undertaken in a laboratory environment with strict speed conditions, compared to the flexible, free-living conditions in our current study. Santos et al.^48^ proposed some new equations to estimate METs with the GT3X accelerometer for youth and adults, yielding RMSEs ranging from 1.20 to 1.55. The RMSE of our model was 1.21 and 1.21 for adults and children, respectively, suggesting good precision of the model. The correlation coefficients between the estimated METs and true METs in previous studies ranged from 0.78 to 0.93 for adults^26,43,44^. In the current study, our results matched that with a Spearman coefficient of 0.87. Moreover, our agreement analysis indicated good ﻿reliability of the estimated METs with 0.89 of ICC and − 0.36 of the mean METs bias.

The wearable smartwatch basing machine learning algorithm provides a more accurate method for measuring PA intensity in comparison with questionnaires. Questionnaires were the most popular measurement for PA in studies of relationship between myopia and PA. However, among these studies, some had found the association between PA and myopia^49–51^, while others had not^5,8^. Many myopia studies used accelerators to measure PA, but most of the devices were placed on the hip of participants^11,12,52,53^, and all the studies measured PA for only 1–2 weeks^11,12,46,52,53^. Compared to a smartwatch, the hip-worn accelerometer was less convenient for long-term daily use. Moreover, the smartwatch has strengths of simple operation, small size, little cost, and more accessible in comparing with the traditional accelerometers. Besides, the smartwatch shows the outdoor time and steps, which contributes to promoting the outdoor activity of children. Conspicuously, there is a noteworthy lack of research that distinguished PA from outdoor time. The confounding protective factors of PA and outdoor time have impeded the understanding of myopia prevention. Notably, our smartwatch could gather data on both outdoor state and PA in real-time. In that way, the effects of outdoor time and PA against myopia could be quantified independently and accurately.

This study developed and validated a new model to classify PA intensities, based on estimated METs for adults and children. The model exhibited excellent accuracy (AUC = 0.96–0.98) for classifying SED and LPA or above and good accuracy for distinguishing MVPA and VPA (AUC = 0.85–0.89) in adults and children. Moreover, the estimated METs ﻿exhibited good accuracy (MAE = 0.75–0.80 METs) and agreement (ICC = 0.84–0.89) when compared with measured METs. Equipped with a highly accurate PA estimation model and an outdoor state discrimination algorithm developed by our team formerly^15^, the smartwatch could be applied to large-scale research, to analyze the role of PA and the outdoor time for myopia protection independently.

Our study aims to apply this model to investigate the relationship between physical activity intensity and the incidence and progression of myopia. The smartwatch developed by our team is equipped with an illuminance sensor and a tri-axial accelerometer, which, along with our previously developed outdoor-indoor state algorithm and the physical activity estimation algorithm from this study, enables us to independently analyze the impact of physical activity on myopia, separate from outdoor time. Furthermore, our smartwatch can potentially be applied to other research areas, such as green space studies, where distinguishing between outdoor activities and overall physical activity is also essential. By enabling such multifaceted applications, our research not only offers a new perspective for understanding and preventing myopia but also lays the groundwork for practical applications of physical activity research in broader contexts.

In this study, we have assessed a diverse population of adults and children aged 9–13, inclusive of both genders, various BMI categories, and a spectrum of physical activities ranging from sedentary to vigorous intensities. Our model has been adjusted for age, gender, and BMI to enhance its applicability across different demographic groups. The data collection was designed to mimic naturalistic settings, incorporating common physical activities such as walking and running, which reflect typical daily exercise patterns. However, the model has its limitations; it was tested and developed exclusively on a Chinese population. Moreover, everyday life activities like writing and sweeping were not included in the data collection, thus limiting its applicability to these types of activities.

There were several limitations in our study. First, although a total of 42 participants completed the activity trial, the sample size for adults and children was relatively small since the children and adults were trained and tested separately in the model. Moreover, the study only focused on children aged 9–13 years and young adults, this results in certain limitations of the model's applicability to a broader range of ages and BMI categories. Accordingly, additional studies are needed to further validate the model with a larger number. Accordingly, additional studies are needed to further validate the model with a larger number of participants. Secondly, PA estimation was less accurate in children than in adults. The reason may be that the sample size of children was smaller than that of adults. Another possible cause might be greater heterogeneity across children compared to adults. Thirdly, although the activity trials were flexible, the accuracy of low-impact free-living activities such as handwriting, sweeping floor, were not evaluated. Nevertheless, the model’s wide spectrum of intensity ranging from SED to VPA may encapsulate the intensity of some of these free-living activities. There are strengths in the study as well. This study estimated the energy expenditure, measured by METs for children from 9 to 13 years old in the first time, whereas the previous study only classified the intensity levels for estimating PA.﻿ In addition, the study expressed the estimated PA intensity as a ﻿numerical variable as well as a ﻿categorical variable. By applying the model, the relationship between PA and myopia can be analyzed both qualitatively and quantitatively.

## Conclusions

Our smartwatch machine learning based model can estimate and classify PA intensity with high accuracy in both adults and children. With the addition of the outdoor state discrimination algorithm, the effect of PA intensity and outdoor time on myopia can be investigated independently by using the smartwatch. So far, the smartwatch has been applied in large-scale research to investigate the relationship between daily activities and myopia^25,54^. Based on the device-based method and large quantity of data, these studies can optimize our understanding of behavioral interventions in myopia onset and progression.

## Data Availability

All data generated or analyzed during this study are included in this published article and its additional information files.

## Abbreviations

PA: Physical activity
EE: Energy expenditure
SVM: Support vector machine
MET: Metabolic equivalent
SED: Sedentary activity
LPA: Light activity
MPA: Moderate activity
VPA: Vigorous activity
MVPA: Moderate and vigorous activity
﻿ROC: Receiver operating characteristic
AUC: Area under the curve
MAE: Mean absolute error
ICC: Intraclass correlation
IQR: Interquartile range
RMSE: Root mean square error
BMI: Body mass index
LoA: Limit of agreement
